# The Multifaceted Impact of Bioactive Lipids on Gut Health and Disease

**DOI:** 10.3390/ijms252413638

**Published:** 2024-12-20

**Authors:** Joseph P. Sullivan, Melissa K. Jones

**Affiliations:** Department of Microbiology and Cell Science, Institute of Food and Agricultural Sciences, University of Florida, Gainesville, FL 32611, USA; josephsullivan1@ufl.edu

**Keywords:** short-chain fatty acids, SCFAs, omega-3 fatty acids, saturated fatty acids, SFAs, sphingolipids, bile acids, inflammatory bowel disease, IBD, colon cancer

## Abstract

Bioactive lipids have a multifaceted role in health and disease and are recognized to play an important part in gut immunity and disease conditions such as inflammatory bowel disease and colon cancer. Advancements in lipidomics, enabled by mass spectrometry and chromatographic techniques, have enhanced our understanding of lipid diversity and functionality. Bioactive lipids, including short-chain fatty acids, saturated fatty acids, omega-3 fatty acids, and sphingolipids, exhibit diverse effects on inflammation and immune regulation. Short-chain fatty acids like butyrate demonstrate anti-inflammatory properties, enhancing regulatory T cell function, gut barrier integrity, and epigenetic regulation, making them promising therapeutic targets for inflammatory bowel disease and colon cancer. Conversely, saturated fatty acids promote inflammation by disrupting gut homeostasis, triggering oxidative stress, and impairing immune regulation. Omega-3 lipids counteract these effects, reducing inflammation and supporting immune balance. Sphingolipids exhibit complex roles, modulating immune cell trafficking and inflammation. They can exert protective effects or exacerbate colitis depending on their source and context. Additionally, eicosanoids can also prevent pathology through prostaglandin defense against damage to epithelial barriers. This review underscores the importance of dietary lipids in shaping gut health and immunity and also highlights the potential use of lipids as therapeutic strategies for managing inflammatory conditions and cancer.

## 1. Introduction

Lipids represent a broad class of hydrophobic molecules categorized based on structure, functional groups, and carbon chain properties. Bioactive lipids are lipids that are capable of affecting cell function via changes in their concentrations. While lipids may have once been considered simply structural components of cellular membranes, the concept of lipids modulating cellular biology has spurred great interest in recent years. This is largely due to improvements in methods of detecting, quantifying, and characterizing lipids and the emergence of the field of lipidomics, which seeks to profile the total lipids in a system, or the “lipidome”. The field of lipidomics has been advanced through methods like mass spectrometry, enabling detailed lipid characterization [1]. Techniques such as electron spray ionization and liquid chromatography allow for efficient separation and identification of lipids, despite challenges related to lipid insolubility in water. These methods have also helped reveal the role of lipids in health, particularly through targeted vs. untargeted analyses, such as those found in the LIPID MAPS database.

A major focus area of lipid research is their interactions in the intestinal tract, which sees a variety of lipids including those made by the host, ingested dietary lipids, and those produced by the gut microbiome. There are several major lipid classes that have been investigated and found to modulate host health and disease. Short-chain fatty acids, such as butyrate, are produced by gut bacteria and play significant roles in immune modulation by suppressing inflammation [2]. In contrast, long-chain saturated fatty acids, like dietary palmitic acid, can induce inflammation, especially in obesity-related conditions [3]. Conversely, unsaturated fatty acids like omega-3s have been shown to reduce inflammation and promote beneficial immune outcomes in diseases [4]. Likewise, fatty acid derivatives, such as prostaglandins, which are generally produced by host cells, can also modulate inflammation and help control immune activity by regulating immune cell behavior [5,6,7]. Though its direct effects on gut immunity are less explored, bile acids—a sterol lipid—modulate immune responses in the gut. Finally, sphingolipids, which are produced by host cells and bacteria, are essential for cell signaling, immune modulation, and membrane structure, and their presence in both eukaryotic and bacterial cells highlights their broad significance. Moreover, the interplay between bacterial sphingolipids and host immune responses implicates their potential as therapeutic targets [8,9,10].

While all the bioactivities of the lipids of the gut are of interest to science, one of the greatest interests is the effects of these lipids in the context of human disease and immunity. For example, many major lipid categories are known to play a role in inflammatory bowel diseases and colon cancer. The complex interactions between dietary lipids, gut-derived lipids, and the microbiome emphasize the role of lipids in maintaining immune balance. Future research will likely uncover more about the lipidome’s impact on systemic health and disease states beyond the gut, such as non-alcoholic steatohepatitis and neurological conditions.

## 2. Lipid Identification

Lipids are a very broad class of hydrophobic/amphiphilic hydrocarbon containing molecules. Lipids have been defined for over 200 years, but due to the complexity of identifying and categorizing these molecules, lipidomics as a field has only evolved over the last two decades [11]. Identification of lipids involves separating molecules into categories based on common structure and origin, resulting in eight main lipid classes. These include (1) fatty acids consisting of elongated carbon chains starting from acetyl-CoA, (2) glycerolipids, which consist of glycerol with one or more fatty acid substitutions often by ester bonds, (3) glycerophospholipids, which are structurally similar to glycerolipids but have a phosphate group substitution on the glycerol head, (4) sphingolipids that originate from a sphingosine base and are formed by the addition of serine to the head of a fatty acid, (5) sterol lipids, which have a distinct four-ring structure, (6) prenol lipids that consist of 5 carbon isoprenoid structures, (7) saccharolipids, which are made of a sugar backbone with fatty acid substitutions, and (8) polyketides, which are defined by alternating ketone and methyl groups. Subcategories of these main lipid classes are based on common modifications to and variants of the head groups as well as subdivisions of lipid biogenesis pathways [12,13].

Specific lipid identification involves separating them into categories based on common structure, then into subcategories based on the number of carbons as well as the bond positions in the lipid tail groups [12,13]. Lipid identification is largely dependent on mass spectrometry (MS), which is a complex process that involves aerosolizing and ionizing the lipid molecules, and then separating the particles/molecules by shooting them through a charged chamber, allowing for separation by mass and charge upon detection [1]. Recent advancements in lipid analysis include the development of targeted vs. untargeted lipidomics. Untargeted methods allow for analysis of the global lipidome while targeted methods rely on the properties of specific lipid categories to separate and further identify specific functional lipid groupings. Lipidomics has also expanded by further characterizing the known categories of lipids, improving detection of smaller concentrations of lipids with more complex mass spectrometry methods [14], and advancing the study of lipids and their function in the context of health and disease [15].

Multiple forms of MS are used to analyze lipids. In all lipid MS applications, the lipids must first be isolated, which requires their separation from the aqueous phase, usually with non-polar solvents. This need for separation presents unique challenges when compared to MS of water-soluble compounds like most metabolites and often requires adaptation of techniques to fit the unique properties of lipids. Even though lipids are not soluble in water, they can still be analyzed by liquid chromatography (LC) prior to MS. This commonly used technique separates categories of lipids prior to MS analysis and allows for broad analysis of complex samples. LC-MS enriches low-concentration lipid categories and can also aid in differentiation of isomeric states of lipids prior to MS [14].

Electron spray ionization (ESI)–MS is another technique commonly used to identify lipids. In this process, a strong electric field is applied to the liquid lipid extract as it slowly passes through a capillary tube, which induces either a positive or negative charge on the lipids prior to their separation by spray through an inert gas [16]. By exploiting the acquisition of either positive or negative charges by different lipid classes, this process enables the separation of lipids by m/z ratio without the need for prior chromatographic separation, which adds significant complexity and possible experimental variation to the process [16]. Removal of LC for lipid analysis makes ESI-MS a faster and less complex technique than LC-MS. However, this comes with the trade-off of reduced sensitivity in samples containing isobaric lipid species or with significant lipid–lipid interactions that may cause difficulties in ionization [16].

Other variations of MS are also used to identify lipid species, and many of these are modifications on the principles of ESI. These include paper spray ionization (PSI), in which the capillary tube of ESI is replaced by a triangular piece of paper with inherent porosity and absorptive properties, making this technique inexpensive and simpler to execute by comparison [17]. PSI also allows for analysis of more crude extracts, including extracts containing particulate matter such as bacterial cultures [18]. Differential mobility separation (DMS)–MS is another alternate technique, which separates charged ions in a gaseous phase in the presence of low- and high-strength electric fields prior to MS analysis. DMS-MS functions similar to ESI-MS but can confer greater sensitivity as it separates molecules with variable electric fields [19]. While the hydrophobic nature and diversity of lipids has made them difficult to properly analyze in the past, this diversity is now being exploited through a variety of mass spectrometry techniques leading to significant gains in the field of lipidomics.

## 3. Lipids Found in the Intestinal Tract

### 3.1. Short-Chain Fatty Acids

One subcategory of lipids that has been studied a great deal in human health is short chain fatty acids (SCFAs, Table 1). These fatty acids are carboxylic acids with small, two to six carbons, aliphatic tails, which are produced by anaerobic bacteria that reside in the gastrointestinal tract and result from bacterial breakdown of certain dietary carbohydrates (i.e., fiber; Ref. [20]). SCFAs, especially butyrate, are major contributors to the health benefits associated with consumption of dietary fiber [2]. Research using wild-type mouse models has shown that butyrate can activate nuclear erythroid 2-related factor 2 (Nrf2), which serves as a master regulator for antioxidant genes leading to a positive correlation with host health [21]. Moreover, in a diabetes animal model, both knockout of Nrf2 and inhibition of co-activator P300 eliminated the benefits of butyrate on aortic oxidative stress and increased aortic damage [21]. These data confirm Nrf2 as a major pathway through which these lipids function and the system-wide benefits of butyrate in both healthy and diseased hosts.

SCFAs have also been associated with a wide range of immune signals in the gut, and commonly function as suppressors of those signals. For example, SCFAs can inhibit the production of both MCP1 and IL10 in human monocytes in the presence of lipopolysaccharide (LPS—another lipid associated molecule with potent immune modulatory effects [22]. SCFAs can also affect the expression of adhesion molecules on leukocytes (L-selectin) and endothelial cells (ICAM-1 and VCAM-1), impacting leukocyte and neutrophil recruitment to sites of inflammation. Although the research on SCFAs’ role in cell recruitment has shown mixed results, this is likely due to the complex nature of the process, which involves multiple signals and cell types. Under inflammatory conditions, SCFAs reduce endothelial adhesion molecule expression [23]. Thus, neutrophil migration and the subsequent exerting an anti-inflammatory effect are decreased. However, SCFAs can also increase L-selectin expression and neutrophil chemotaxis under some circumstances [24] while lowering expression pro-inflammatory cytokines such as *tnfα*, *cinc2α/*β, and *nfkβ* under other conditions [25]. This leads to varied effects on inflammation. One important caveat is that these studies were performed using mouse models of disease, and their translational relationship to human disease is in question. Ultimately, SCFAs play a significant, condition-dependent role in modulation host inflammation in the gut via leukocyte recruitment.

SCFAs have also been shown to improve the function and homeostasis of the intestinal barrier. For example, propionate given to murine intestinal epithelial cells (IECs) in cultures was shown to promote the migration of the IECs through enhanced cell polarization and actin remodeling [26]. In epithelial organoid models, it has been shown that acetate treatment decreases expression of inflammatory cytokines further supporting the positive impact of SCFAs on intestinal health [27]. On a more general level, the impact of SCFAs extends to modulating the gut microbiota composition. A balanced microbial community is essential in preventing dysbiosis, a common feature in IBD that leads to reduced SCFA production and plays a significant role in cancer prevention. High levels of SCFAs promote the growth of beneficial bacteria, which further produce anti-inflammatory molecules, creating an environment that is less conducive to cancer development and can resist pathogenic bacteria, which can alleviate IBD symptoms [28,29].

### 3.2. Eicosanoids

Another type of lipid that is shown to have anti-inflammatory effects are eicosanoids. These are a family of immune regulating poly-unsaturated fatty acids produced by mammals. These lipids contain the well-known prostaglandins and other derivatives of arachidonic acid and similar unsaturated fatty acids (Table 1). Prostaglandins are generally thought to be pro-inflammatory as they are associated with inflammation and help to regulate blood flow and pain sensation [30]. However, their effects are likely determined by the context in which these lipids are being produced. One prostaglandin found in the gut, prostaglandin E2 (PGE2), is known to prevent innate immune responses by binding to the EP4 receptor. This activity makes it one of the many tolerogenic signals that allows for the maintenance of the gut microbiome [31]. PGE2 may also reduce the production of inflammatory TNFα while increasing the production of anti-inflammatory IL-10 leading to a localized reduction in inflammation [32]. Another variety of eicosanoids are the specialized pro-resolving lipid mediators (SPMs). These lipids can inhibit the production of inflammatory eicosanoids while increasing the production of more SPMs [33]. SPMs also have some control over the maturation of antigen presenting dendritic cells and the cytokine production of T cells, preventing the maturation of cytotoxic T cells [33]. These complex fatty acids have a great deal of importance in regulating immune responses. In fact, these lipids play a significant role in the inflammatory mechanisms of IBD (particularly ulcerative colitis and Crohn’s disease), colon cancer, and diverticulitis [34,35].

### 3.3. Dietary Lipids

In addition to SCFAs and eicosanoids, other dietary lipids have been associated with immune modulation in the intestinal tract (Table 1). The term “fat” is generally used to refer to dietary lipids, which includes saturated and unsaturated fatty acids. Saturated fatty acids (SFAs) have no double bonds in the carbon backbone and are common in animal meats and the western diet in general. SFAs are not their own class of lipid and can generally be free fatty acids (like the SCFAs) or associated with other molecules like glycerol as in the case of mono-, di-, and triacylglycerols, which are part of the glycerolipids category of lipids [12]. SFAs and especially triacylglycerol (triglyceride) are the main components of high-fat diets [36], and heavy consumption of these lipids are generally associated with negative health effects. For example, mice fed a diet of saturated fats had increased sensitivity to LPS and increased mortality [3]. This same study also found that pre-treating mice with palmitic acid resulted in hyper-inflammatory responses by macrophages and that this response was mediated by ceramides (another type of lipid discussed below).

The saturated fat palmitic acid has also been shown to increase the production of pro-inflammatory cytokines MCP1, IL-6, and IL-8 in macrophages, as well as attracting neutrophils [37]. The induction of these cytokines in macrophages and dendritic cells is thought to result from palmitic acid functioning as a TLR4 agonist. This ultimately leads to activation of the NLRP3 inflammatory pathway to release these cytokines downstream [38,39]. In fact, the TLR4 agonist theory helped explain how the long-chain SFAs could mediate the low-grade, persistent inflammation seen in obesity and associated conditions. However, work by Lancaster et al. [40] found that long-chain fatty acids still induced inflammatory responses in macrophages even in a TLR4 knockout, but only if other toll-like receptors were activated first. This suggested that long-chain fatty acids function as a “second hit” to activate inflammatory responses [40]. This finding is especially interesting in the context of the gut, since LPS (a canonical TLR4) agonist, is a ubiquitous component of gram-negative bacteria and thus found throughout the intestinal tract. Furthermore, in addition to macrophages and dendritic cells, palmitic acid also induces pro-inflammatory phenotypes in T cells [41]. Altogether, this ability to induce pro-inflammatory states in a wide array of immune cell types highlights how SFAs can modify the immune system in the gut and contribute to chronic inflammation without having to directly induce the inflammatory environment.

Unlike saturated fatty acids, some unsaturated fatty acids, which are acquired from dietary intake of oils from plants or fish, are viewed as healthy components of the diet. Unsaturated fatty acids have at least one double bond in their carbon chain, and a primary example of the beneficial effects of these lipids is the activity of omega-3 fatty acids. Omega-3s have been shown to help reduce intestinal inflammation in both healthy and disease conditions [4]. It is thought that omega-3 fatty acids function as competitors for the conversion of arachidonic acid into prostaglandins and leukotrienes. Thus, limiting the production of omega-3s from arachidonic acid leads to a reduction in inflammation [4]. Omega-3 fatty acids also demonstrate their beneficial effects through modulation of cytokine expression and chemotaxis [42]. For example, docosahexaenoic acid (DHA) has been shown to increase M2 polarization in macrophages [43], and the M2 activation state is an anti-inflammatory and immunoregulatory phenotype [44]. In addition, DHA decreases expression of pro-inflammatory genes in macrophages [43]. These findings highlight the potential of omega-3 fatty acids as powerful dietary tools for modulating inflammation and promoting immune balance.

### 3.4. Glycerophospholipids

Glycerophospholipids are one of eight major lipid classes. These lipids are composed of fatty acid chains linked to a glycerol by ester linkages, and a phosphate group associated with the glycerol (Table 1). Glycerophospholipids are the major class of lipids in biological membranes, and their structure is largely the same between bacteria and eukaryotes [45,46,47]. There are slight differences between the kingdoms due to the structure of the fatty acid chains, where bacteria may produce shorter fatty acid chains or have different double bond positions [48]. The greatest difference in glycerophospholipid structure is in those produced by archaea. Archaeal glycerophospholipids utilize isoprene chains rather than fatty acids and are linked by ether linkages to L-glycerol rather than the D stereoisomer found in bacteria and eukaryotes [49]. These differences are likely due to the different requirements of cellular membranes across these domains.

Despite their structural and functional importance across all domains of life, research on the health impacts of glycerophospholipids is limited. Findings, such as changes to their metabolism being observed in models of depression [45,50,51,52,53], have been reported. In fact, their extreme ubiquity, making up most of the membrane of most cells, can make it difficult to study the effects of these lipids. For example, experiments involving deletion or extreme modification of these lipids are not possible as they are often lethal to the organism. Their ubiquity also makes glycerophospholipids a poor choice for receptor binding, as receptors would be saturated in most situations. Yet, having these lipids readily available in cell membranes, where receptors are often found, does open up their uses as signaling molecules. For example, it is more favorable for an organism to use a modified membrane lipid to signal receptor activation than to produce a new molecule for signaling receptor activation. Overall, these characteristics underscore the dual role of glycerophospholipids as essential structural components and versatile signaling molecules, highlighting their significance in both cellular functionality and potential health implications regardless of the challenges in studying their specific effects.

### 3.5. Bile Acids

Cholesterol, which is a sterol lipid, is a well-known molecule of interest in the context of health, possibly best known for its contribution to heart and circulatory diseases, but also of critical function in cell membranes. There is little research showing an impact of cholesterol on gut immunity. However, other sterol lipids (which are derived from cholesterol metabolism) are known to modify immunity and potentially the gut microbiota [54]. One example is bile acids, which are the main component of fat-digesting bile that is released into the small intestine (Table 1). Research on the regionalization of norovirus infection within the gut has shown that microbiome-dependent regionalization of infection in the gut is largely due to bile acid, in which host-derived bile acids promote a pro-inflammatory environment, while those modified by gut bacteria mediate anti-inflammatory effects [55]. During times of disease and inflammation, bile acid metabolism is often disrupted, which ultimately leads to additional inflammatory potential [56]. Moreover, bile acids also interact with the intestinal microbiome and can shape the bacterial composition by promoting or inhibiting the growth of select bacterial species [56]. While research into the effects of sterol lipids on the immune responses of the gut still needs to be developed, these lipids and their derivatives are among the most significant bioactive molecules in immune regulation and beyond.

### 3.6. Sphingolipids

Another class of bioactive lipids that have been of significant interest are sphingolipids. These are defined by a sphingosine backbone and contain associated groups and residues that give each of them a specific subclass and structure (Table 1). Sphingolipids contain a long chain of carbons that are associated with a hydrophilic head group. Many sphingolipids also contain a fatty acid residue. When this is the only addition to the sphingosine head, then the lipid is referred to as a ceramide. Since their discovery in 1870, these lipids have been associated with a variety of functions, including use in cellular membrane domains, cell signaling, cell proliferation, cell death, cell migration and invasion, central nervous system development, and immunomodulation [57,58,59]. In fact, sphingolipids are essential for the maintenance of most eukaryotic cell membranes, although they have traditionally been observed within the context of the nervous system [58,60].

One of the more studied functions of sphingolipids in immunomodulation is the trafficking of immune cells. This includes T cells, which are often needed to clear viral and bacterial infections. To perform this function, T cells need to enter circulation after exiting the lymph nodes. Sphingosine 1 phosphate (S1P) has been observed to play a major role in this process. This process benefits from the presence of an S1P gradient between circulation and tissue, in which there is a greater expression of S1P in circulation [50]. Moreover, when hematopoietic cells are unable to express the receptor S1PR, T cell egress from lymph nodes is significantly reduced [61]. Sphingolipids are also involved in immune processes in macrophages. For example, the stimulation of TLR2 and TLR4 by bacterial lipopeptides and LPS induces S1P production by upregulating expression of its activating kinase [62,63]. It is suggested that S1P interaction with its receptor (S1PR3) contributes to the production of the chemokine MCP1, which in turn recruits monocytes to the site of infection [62]. Stimulation of another S1P receptor (S1PR2) has also been shown to induce expression of inflammatory molecules IL-1β and IL-18 in mice [64]. These findings, among others, demonstrate the critical role of sphingolipids in immune cell trafficking and inflammatory regulation, emphasizing their importance as mediators of the immune response and pointing to their involvement in inflammatory diseases.

### 3.7. Bacterial Sphingolipids

Sphingolipids were first described and initially known only to exist in eukaryotic organisms. However, in recent years, production of these lipids has been observed in prokaryotes. Structural differences exist between bacterial and eukaryotic sphingolipids, which may be due to differences in associated head groups or fatty acids, similar to in glycerophospholipids. However, one primary difference that makes eukaryotic sphingolipids distinguishable from bacterial sphingolipids is that eukaryotic lipids have 18 carbons with a single double bond in the sphingolipid base [45]. Sphingolipid production has been best described for the phyla *Bacteroidetes* and *Proteobacteria*; however, prokaryotes outside of these phyla may also produce sphingolipids [57,58,60,65]. The most studied source of bacterial sphingolipids in the gut are members of the *Bacteroidetes* phylum, including *Bacteroides thetaiotaomicron* (which is one of the most abundant bacterial groups in the human gut microbiome). Lipidomic analysis of *B. thetaiotaomicron* has shown that sphingolipids are one of the most abundant lipid classes in their membranes [66]. The same has also been shown for the lipid content of outer membrane vesicles (OMVs) produced by this bacterium [60].

Like eukaryotic sphingolipids, bacterial sphingolipids also have immune modulatory capabilities. In germ-free mice that were monocolonized with either wild-type or a sphingolipid knockout strain of *B. thetaiotaomicron*, a significant elevation in IL-6 and MCP1 levels and crypt hyperplasia were observed in mice colonized with the sphingolipid mutant [45]. Moreover, it was also shown that host-derived sphingolipids were increased in IBD patients and positively correlated with inflammation and those derived from *Bacteroides* were decreased and negatively correlated with inflammation [45]. While the mechanism by which *Bacteroides* sphingolipids modulate host inflammation is still unclear, it has been suggested that these lipids may have a role in tolerogenic responses in T cells [45]. Taken together it may be that as bacterial-derived sphingolipids are decreased due to dysbiosis, host-derived sphingolipids increase, and this dysregulation leads to inflammation. The inflammation may be a result of increased immune cell trafficking toward sphingolipids like S1P, or it may be a result of the loss of tolerogenic signals from bacterial sphingolipids to regulatory T cells, but this area requires more study.

Both bacterial sphingolipids and SCFAs have been shown to reduce the expression of MCP1 among other inflammatory signals. The opposite has been shown of other lipids, such as the long-chain fatty acid, palmitic acid. Taken together, the propensity for lipids to modulate immune signaling in the gut suggests that the gut microbiome and diet, by way of lipids, help mediate inflammation and that modification to lipid profiles of immune cells may also induce inflammation.

## 4. The Role of Lipids in Intestinal Disease

### 4.1. Inflammatory Bowel Disease

Lipids play diverse roles in modulating inflammatory and metabolic diseases through their ability to mediate inflammation. While some lipids exacerbate inflammation and disease, others reduce them and thus aid in prevention and resolution of disease. Among the lipids that reduce inflammation, SCFAs are crucial in managing IBD conditions such as Crohn’s disease and ulcerative colitis (Table 2). Molecules such as butyrate, acetate, and propionate function as an energy source for colon cells and promote a healthy gut barrier. Butyrate, for instance, strengthens colonocytes, which helps maintain the integrity of the intestinal barrier [67], preventing harmful substances from crossing into the bloodstream—a critical factor in managing IBD symptoms [68].

#### 4.1.1. Bioactive Lipids Associated with Reducing Intestinal Inflammation

As discussed above, SCFAs have anti-inflammatory properties, which are crucial for reducing the immune response associated with disease (Table 2). SCFAs stimulate the activity of regulatory T cells (Tregs—immune cells that help prevent excessive inflammation) through pathways involving G-protein coupled receptors such as FFAR2 (GPR43) and HCAR2 (GPR109A) on dendritic cells (DCs) and macrophages. This activation triggers IL-10 production, which facilitates Treg cell differentiation and ultimately aids in maintaining gut homeostasis and reducing inflammatory responses [69,70]. Through these mechanisms, SCFAs reduce inflammation and improve gut health. Moreover, murine studies have demonstrated that supplementation with butyrate and other SCFAs enhance Treg function and subsequently lower inflammation [71]. In addition, clinical interventions focusing on dietary fiber intake and probiotic interventions to boost SCFA production have shown promise in supporting gut health and reducing the severity of IBD, emphasizing the importance of SCFAs in disease management [71,72]. This indicates that increasing dietary SCFA levels shows promise in IBD management.

In addition to the direct effects on immune modulation of disease, SCFAs also exert cellular effects (Table 3). One way through which this occurs is via epigenetic mechanisms, notably by inhibiting histone deacetylases (HDACs). HDACs enhance the expression of genes associated with anti-inflammatory pathways. HDAC inhibition by SCFAs promotes the acetylation of histones, leading to the activation of FoxP3+ Tregs. For instance, butyrate enhances the production of Treg cells in the colon by directly increasing the expression of FoxP3 through histone acetylation at conserved sequences, thus contributing to a reduced inflammatory state [73]. These molecular changes bolster the production of IL-10, further supporting Treg cell function and mitigating inflammation in IBD [71,74]. Interestingly, HDAC inhibition by SCFAs may also exert control epithelial barrier integrity. Using murine IECs in vitro, propionate treatment showed that HDAC inhibition was directly related to improved IEC migration, which is associated with improved barrier integrity [26].

Additionally, SCFAs indirectly promote Treg activity by modulating metabolic pathways within T cells. Butyrate increases glycolysis and mitochondrial activity, providing the necessary energy and substrates for Treg proliferation and function. By acting as a substrate for acetyl-CoA in T cells, SCFAs also enhance their metabolic flexibility, enabling them to respond efficiently to inflammatory signals. This metabolic support underlies SCFAs’ role in maintaining an anti-inflammatory environment in the gut, highlighting their potential as therapeutic agents in IBD [75]. Overall, these findings suggest that increasing SCFA levels, either through dietary interventions or targeted therapies, may be beneficial for IBD patients.

Like SCFAs, some omega-3 fatty acids (e.g., EPA and DHA) also exhibit anti-inflammatory effects and have shown potential in managing IBD [76]. Interestingly, these fatty acids modulate inflammation in the gut by altering cell membrane composition. The incorporation of EPA and DHA into cell membranes reduces the presence of arachidonic acid, which is a precursor to pro-inflammatory eicosanoids (Table 3). By shifting this balance, omega-3s decrease the production of inflammatory molecules and increase the synthesis of anti-inflammatory mediators, which help resolve inflammation [76,77,78]. Mechanistically, omega-3 fatty acids influence gene expression through nuclear receptors such as peroxisome proliferator-activated receptor gamma (PPARγ; Table 2). When activated by omega-3s, PPARγ reduces the expression of pro-inflammatory cytokines, including TNF-α and IL-6, both of which are elevated in IBD. This modulation of gene expression helps in reducing inflammation in the gut mucosa and supports tissue healing, making omega-3 fatty acids beneficial as a dietary supplement in managing IBD flare-ups [77,79].

#### 4.1.2. Bioactive Lipids Associated with Inducing Intestinal Inflammation

In contrast to SCFAs and omega-3 fatty acids whose presence leads to improved barrier integrity and reduction of IBD symptoms, saturated fatty acids promote intestinal inflammation and disruption of gut homeostasis. Thus, these lipids can contribute significantly to the development and exacerbation of IBD (Table 3). High saturated fatty acid (SFA) intake can lead to an altered gut microbiome, favoring the growth of pro-inflammatory bacteria while reducing beneficial species [80]. This microbial dysbiosis enhances the intestinal inflammatory response, which is central to IBD pathology. The resulting inflammation can reduce gut barrier integrity and aggravate IBD symptoms [72,81].

TLR4 is one of the primary pro-inflammatory pathways activated by SFAs within the gut [82] (Table 4). Stimulation of TLR4 by SFAs triggers the release of TNF-α and IL-1β which can lead to the chronic inflammatory state characteristic of IBD [83]. Studies have noted that elevated TLR4 signaling due to high SFA intake is associated with increased disease severity in Crohn’s disease and ulcerative colitis patients [84].

In addition, SFAs induce oxidative stress in the intestinal lining, leading to damage of cellular structures and DNA. This oxidative stress occurs when SFAs increase the production of reactive oxygen species (ROS), causing damage to intestinal cells and deterioration of the gut barrier [85]. This compromised barrier not only allows pathogens to enter but also maintains an inflammatory cycle that perpetuates tissue damage. For IBD patients, this oxidative stress is particularly detrimental, as it contributes to the progression of inflammation and increases disease severity [86]. Moreover, SFA-rich environments are linked to impaired Treg function, which leads to uncontrolled inflammation and exacerbation of IBD [87]. Therefore, diets high in SFAs contribute to the persistence and severity of IBD, which also underscores the importance of dietary management in these affected individuals [28,72].

Like SFAs, the poly-unsaturated eicosanoids also drive inflammation but do so through differing pathways that involve cyclooxygenase (COX) and lipoxygenase (LOX) enzymes (Table 3). The COX-2 enzyme produces high levels of PGE2, which acts on receptors in immune and epithelial cells to promote inflammation, increase vascular permeability, and recruit immune cells to the gut lining [88,89]. This process sustains the chronic inflammatory environment that is characteristic of IBD, contributing to tissue damage and exacerbating disease symptoms.

Leukotrienes, produced by the LOX pathway, also contribute to IBD pathology by attracting immune cells like neutrophils to inflamed areas in the intestines (Table 4). Leukotriene B4 amplifies the inflammatory response by binding to its receptors on immune cells, which promotes further cytokine release and intensifies gut inflammation. Moreover, PGE2 and leukotrienes also activate NF-κB amplifying inflammatory gene expression and leading to sustained inflammation [90]. This leukotriene-driven recruitment of immune cells is especially problematic in Crohn’s disease, where it contributes to deep tissue damage and may worsen disease severity [5].

Eicosanoids also play a role in altering the epithelial barrier integrity in IBD. Eicosanoid induction of PGE2 and thromboxanes can disrupt tight junctions between epithelial cells, making the intestinal barrier more permeable and contributing to the “leaky gut” phenomenon observed in many IBD patients (Table 3). This compromised barrier function exacerbates inflammation and increases the risk of infection, highlighting the role of eicosanoids in disease progression [88,89].

Sphingolipids are also associated with IBD; however, their role in disease is more complex. Some sphingolipids reduce inflammation and colonic damage, while others mediated immune cell trafficking and inflammation. To further complicate the role of sphingolipids in disease, those produced by the gut microbiota appear to modulate inflammation, suggesting a potential therapeutic avenue for these lipids. The involvement of sphingolipids in IBD was first considered due to the association between TNFα (Table 4; a common target in IBD therapies) signaling and sphingolipids [91]. It was also found that sphingolipids are one of the most differentially abundant metabolites in IBD patients compared to healthy patients [92].

Based on these observations, further studies using dextran sulfate sodium-induced colitis in mice were performed and showed that a sphingomyelin analog significantly reduced colonic damage and cytokine production associated with colitis [93]. This was thought to result from the sphingomyelin leading to a reduction in ceramide production, which was shown to increase colonic cell viability [93]. In an IL10^−/−^ mouse model of IBD, another S1PR agonist, KRP-203 was shown to reduce TH1 response and colitis, which was associated with T cell trafficking [94] and linked to previously identified effects of S1P on T cells [50] (Table 2). However, it should be noted that KRP-203 was tested for efficacy as an IBD treatment and did not pass clinical trials [95]. Taken together, sphingolipids may have paradoxical effects in the gut, both decreasing inflammation and increasing it in models of colitis. This becomes more interesting when exploring the effects of sphingolipids derived from the gut microbiome.

### 4.2. Colon Cancer

Colon cancer development is influenced by a complex interplay of dietary, microbial, and host-derived lipids, which can either promote tumorigenesis or provide protective effects through immune and metabolic modulation. As with IBD, protective lipids include SCFAs and omega-3 fatty acids. These lipids share some overlapping mechanisms with IBD that are also protective against cancer. One example is the ability of butyrate to serve as an energy source for colonocytes, strengthening the cells lining the colon, and thus providing a protective role against colon cancer (Table 3). However, these lipids also have cancer-specific effects. For example, the ability of butyrate to inhibit HDACs leading to changes in gene expression that suppress cancer cell proliferation and promote apoptosis in cancerous cells, highlighting its potential as a therapeutic agent [69,96].

Beyond epigenetic regulation, SCFAs impact cellular metabolism through the mTOR pathway, further contributing to tumor suppression by enhancing autophagy. SCFAs can suppress mTOR activity through a long non-coding RNA (RMST) activated pathway, which ultimately enhances autophagy [97,98]. Autophagy induced by SCFAs prevents the accumulation of cellular damage and supports normal cell function, while limiting the growth of cancerous cells [28,99]. SCFAs can also contribute to immune regulation in the colon, influencing inflammatory responses that are often implicated in colorectal cancer. By activating receptors such as GPR43 (FFAR2) and GPR109A, SCFAs enhance the release of anti-inflammatory cytokines, including IL-10, to aid in maintaining homeostasis (Table 2). This reduction in inflammation can limit the conditions that promote tumor formation.

Additionally, SCFAs play a role in maintaining gut barrier integrity through mechanisms that reduce oxidative stress and inflammation, which are both risk factors for cancer development [97,98]. Epigenetically, SCFAs influence DNA methylation and histone modification processes, which are essential for regulating gene expression in colon cells. The inhibition of HDACs by butyrate leads to increased acetylation of histones, thereby promoting the expression of tumor suppressor genes (Table 3). This epigenetic modulation by SCFAs results in decreased tumor growth and improved cancer cell apoptosis rates. Studies have demonstrated that diets high in fiber, which increase SCFA production, are associated with lower rates of colon cancer, emphasizing the potential of dietary interventions as preventative measures [28,97]. Thus, like IBD, it is the presence of SFCAs and the anti-inflammatory impact of these lipids that can potentially prevent the onset and progression of disease.

Interestingly, omega-3 fatty acids can impact colon cancer through altering cancer cell metabolism (Table 3). Cancer cells rely heavily on lipid synthesis for growth and survival, and omega-3s can interfere with this process by modulating enzymes involved in lipid metabolism. For example, they reduce the expression of fatty acid synthase, which is an enzyme often overexpressed in colorectal cancer that supports cancer cell proliferation. By downregulating this enzyme, omega-3s can inhibit cancer growth and make cells more susceptible to apoptosis, thereby slowing cancer progression [100,101]. Omega-3 fatty acids also enhance the effectiveness of conventional cancer therapies like chemotherapy and radiation by increasing oxidative stress within cancer cells. This selective increase in oxidative stress renders cancer cells more vulnerable to treatment, enhancing therapeutic outcomes. Moreover, the anti-inflammatory and immunomodulatory effects of omega-3s help mitigate some of the inflammatory side effects associated with these treatments, providing additional support for patients undergoing cancer therapy [102,103].

In contrast to the protective effects of SCFAs and omega-3 fatty acids, saturated fats and pro-inflammatory eicosanoids exacerbate cancer progression by promoting chronic inflammation and oxidative stress (Table 3). In addition to inducing inflammation, saturated fatty acids (SFAs) also interact with lipid metabolic pathways that are often dysregulated in cancer. High levels of SFAs in the diet can elevate expression of enzymes like SCD1, which converts SFAs into monounsaturated fatty acids (MUFAs). SCD1 overexpression has been correlated with poor cancer prognosis, as it enhances cancer cell survival and resistance to apoptosis [104].

By promoting the synthesis of MUFAs, SFAs indirectly support lipid signaling and membrane synthesis crucial for rapid cancer cell proliferation. The upregulation of SCD1 and its associated pathways highlights how dietary SFAs may stimulate lipid-dependent mechanisms, exacerbating cancer progression [104,105]. Additionally, SFAs influence oxidative stress within the colon, which can damage DNA and lead to mutations, further contributing to cancer risk. Reactive oxygen species (ROS), which are produced as a byproduct of high SFA levels, cause oxidative damage in cellular structures and DNA. This oxidative stress, combined with the inflammatory environment created by SFA-induced TLR activation, fosters a cycle of DNA damage and cell proliferation, increasing the likelihood of malignant transformations in colon cells [29]. Together, these findings highlight the dual role of lipids in colon cancer, with dietary interventions targeting SCFAs and omega-3 fatty acids offering promising therapeutic potential to mitigate tumor progression and improve patient outcomes.

## 5. Conclusions

A healthy gut microbiome and lipidome inhibit inflammation, and an aberrant one can lead to inflammation and disease. Advancements in mass spectrometry have revolutionized the field of lipid research, enabling more precise identification and characterization of diverse lipid species. This has ultimately expanded our understanding of bioactive lipids and their diverse roles in biological processes and disease. Techniques such as LC-MS, ESI-MS, and their variations have addressed the challenges posed by the hydrophobic nature and structural diversity of lipids, allowing for both targeted and untargeted analyses. These innovations have significantly expanded our understanding of lipids, their biogenesis, and their roles in health and disease, marking a new era in lipid research.

**Figure 1:**
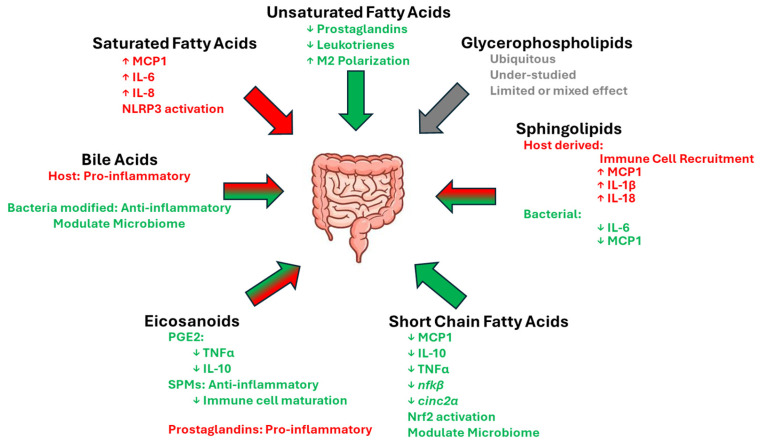
Overview of positive (green arrows and text), negative (red arrows and text), and neutral (grey) impacts of dietary lipids and their associated immune modulators.

Among the most studied bioactive lipids, SFCAs have been proven to play a pivotal role in gut health, acting as key modulators of inflammation and contributors to microbiota balance (Figure 1). Their ability to suppress inflammatory signals, regulate leukocyte recruitment, and promote the growth of beneficial bacteria highlights their broad impact on both immune function and disease prevention. These findings emphasize the therapeutic potential of dietary fiber and probiotics in boosting SCFA production to support gut health and manage conditions such as IBD and metabolic disease. Moreover, recent research has shown that SCFAs have benefits beyond gut health. For example, murine models have shown these fatty acids can reduce inflammation in non-alcoholic steatohepatitis [106,107]. In addition, their ability to improve gut barrier integrity is also linked to improved outcomes of pancreatitis in mouse models [108]. Future research exploring the systemic impact of SCFAs will likely reveal additional benefits in host health and remediation of disease.

Another critical regulator of immune responses are eicosanoids, with their effects largely determined by the context in which they are produced. Prostaglandin E2 and SPMs exemplify the anti-inflammatory potential of these lipids, highlighting their role in maintaining gut homeostasis and reducing localized inflammation (Figure 1). These findings underscore the importance of eicosanoids in modulating inflammatory conditions such as IBD, colon cancer, and diverticulitis.

Dietary lipids are also heavily involved in shaping immune responses in the gut, with saturated and unsaturated fatty acids exerting contrasting effects. Saturated fats, such as palmitic acid, are associated with pro-inflammatory states, contributing to chronic inflammation through mechanisms involving toll-like receptor activation and inflammatory cytokine production (Figure 1). Conversely, unsaturated fatty acids, particularly omega-3s, demonstrate anti-inflammatory properties by modulating cytokine expression and promoting immune-regulatory phenotypes like M2 macrophage polarization. These data also highlight the therapeutic potential of unsaturated fatty acids and SCFAs in managing inflammation and promoting overall health. Future research investigating the direction interaction of omega-3s with nuclear receptors, transcription factors, and signaling cascades that are specific to intestinal immunity would further clarify their role of omega-3 fatty acids in gut health.

The less heavily researched glycerophospholipids serve as both indispensable structural components of cellular membranes and versatile signaling molecules, emphasizing their critical role in cellular functionality. Despite their ubiquity posing challenges for targeted research, their involvement in signaling and metabolism underscores their potential health implications. These dual roles (Figure 1) highlight the need for innovative approaches and future research to further understand their complex biological functions. Likewise, with both pro- and anti-inflammatory effects, bile acids, depending on their source demonstrate their ability to interact with and influence the microbiome. These findings highlight the complex role of sterol lipids in immune regulation and the need for further investigation into their functions in gut health.

Likewise, sphingolipids play a pivotal role in cellular and immune processes, serving as key mediators in immune cell trafficking and inflammatory regulation (Figure 1). Their involvement in T cell egress, macrophage activation, and chemokine production demonstrates their importance in maintaining immune homeostasis and responding to infections. The ability of sphingolipids like S1P to influence inflammatory pathways highlights their dual role in promoting immune responses and contributing to inflammatory diseases. These findings emphasize the need for further exploration into sphingolipid-mediated mechanisms as potential therapeutic targets in immune-related conditions.

In conclusion, bioactive lipids are instrumental in shaping immune responses. Short-chain fatty acids like butyrate exhibit anti-inflammatory effects, whereas long-chain saturated fats, such as palmitic acid, often induce inflammation. The interplay between dietary lipids, immune modulation, and gut health underscores their significance in chronic conditions such as IBD, metabolic disorders, and obesity. Meanwhile, unsaturated fats, particularly omega-3 fatty acids, offer protective effects by reducing inflammation and promoting immune balance, suggesting dietary interventions such as modulation of lipid consumption could be employed as a therapeutic strategy for improving gut health and preventing or resolving chronic disease.

## Figures and Tables

**Table 1:** Major lipid categories found in the intestinal tract and specific lipids within each category that have identified roles in regulating host heath and disease.

Major Lipid Categories	Specific Lipids
Short-Chain Fatty Acids	Butyrate
	Acetate
	Propionate
Eicosanoids	Prostaglandins (e.g., PGE2)
	Specialized Pro-Resolving Mediators (SPMs)
Dietary Lipids	Saturated Fatty Acids (e.g., Palmitic Acid)
	Unsaturated Fatty Acids (e.g., Omega-3s like EPA and DHA)
Glycerophospholipids	Phosphatidyl inositol
	Phosphatidic acid
	Phosphatidyl ethanolamine
	Phosphatidic choline
	Phosphatidyl serine
Bile Acids	Cholic acid
	Deoxycholic Acid
	Lithocholic Acid
	Taurocholic Acid
Sphingolipids	Ceramides
	Sphingosine-1-Phosphate (S1P) Bacterial derived sphingolipids

**Table 2:** Lipids and associated immune factors that counteract intestinal disease.

Disease Name	Disease Inhibiting Lipids	Regulated Immune Factors
IBD	Short-Chain Fatty Acids (e.g., Butyrate, Acetate, Propionate)	Stimulate Treg activity
		GPR43/HCAR2 upregulation IL-10 production Stimulation of FoxP3+ Tregs Reduced NF-kB activity
	Omega-3 Fatty Acids (e.g., EPA and DHA)	Reduces TNFα and IL-6 expression IL-10 induction M2 macrophage polarization Reduced COX-2 activity
	Sphingolipids	Reduction of TNF-α Reduce TH1 response IL-10 induction
Colon Cancer	Short-Chain Fatty Acids (e.g., Butyrate)	Tregs proliferation
		Enhanced FoxP3 expression Activate GPR43 and GPR109A
		IL-10 induction
	Omega-3 Fatty Acids (e.g., EPA and DHA)	PPARy activation

**Table 3:** Cellular effects of bioactive lipids.

Lipid Type	Cellular Effects in Promoting Disease	Cellular Effects Inhibiting Disease
Short-Chain Fatty Acids	N/A	Epigenetic HDAC inhibition Increase glycolysis Increase mitochondrial activity Energy source for healthy cells Promote cancer cell apoptosis Suppress tumor proliferation Alter cell metabolism via mTOR Enhance autophagy Reduce oxidative stress
Saturated Fatty Acids	Promote oxidative stress Elevate SCD1 expression Enhance cancer cell survival Increased apoptosis resistance ROS production Promote DNA damage	N/A
Omega-3 Fatty Acids	N/A	Alter cell membrane composition Reduces arachidonic acid levels Influence expression of PPARγ Reduce fatty acid synthase to inhibit cancer growth Increase oxidative stress in cancer cells
Eicosanoids	COX-2 pathway induction LOX pathway induction Disrupt tight junctions Increases inflammatory leukotriene production	Can inhibit inflammatory eicosanoids
Sphingolipids	Reduce colonic cell viability	Reduce colonic cell damage

**Table 4:** Lipids and associated immune factors that promote intestinal disease progression.

Disease Name	Disease Promoting Lipids	Regulated Immune Factors
IBD	Saturated Fatty Acids (e.g., Palmitic Acid)	TNF-α and IL-1β
		TLR4 activation Impaired Treg function
		NF-κB
	Eicosanoids (e.g., PGE2, Leukotrienes)	Neutrophil recruitment Activate NF-κB
	Sphingolipids (e.g., Ceramides)	MCP1 activation TLR2/4 activation IL-1β IL-18
Colon Cancer	Saturated Fatty Acids (e.g., Palmitic Acid)	TNF-α
		IL-1β
		ROS production
		TLR4 signaling NF-kB Oxidative-stress induced DNA damage
